# Temporal dynamics and *Leishmania infantum* infection prevalence of *Phlebotomus perniciosus* (*Diptera*, *Phlebotominae*) in highly endemic areas of visceral leishmaniasis in Tunisia

**DOI:** 10.1371/journal.pone.0184700

**Published:** 2017-09-21

**Authors:** Meriem Benabid, Jamila Ghrab, Adel Rhim, Rania Ben-romdhane, Karim Aoun, Aïda Bouratbine

**Affiliations:** 1 Department of Parasitology, Research Lab: LR 11-IPT-06, Pasteur Institute of Tunis, University Tunis El-Manar, Tunis, Tunisia; 2 Department of Biotechnology, Higher Institute of Sciences and Technology of Environment of Borj Cedria, University of Carthage, Tunis, Tunisia; University of Ostrava, CZECH REPUBLIC

## Abstract

*Phlebotomus perniciosus* is one of the major vectors of *Leishmania infantum* in the Mediterranean basin. The aim of this work was (i) to provide information about abundance and temporal dynamics of this *Larroussius* species in a hot spot area of visceral leishmaniasis in Tunisia, (ii) to detect *L*. *infantum* DNA in wild caught female sandflies and (iii) to measure *Phlebotomus perniciosus* infection rate throughout the active season. Sandflies were collected monthly during one year using CDC miniature light-traps in house and in animal shelters. Male specimens were identified at species level according to morphological characters. Female specimens were conserved individually for molecular study. *Leishmania* infection was tested by kinetoplast DNA real-time PCR and ITS-1 PCR-sequencing. Subsequent sandfly species identification of infected specimens was done by mitochondrial cytochrome b sequencing. In one year period, overall 4,441 specimens (2230 males and 2211 females) were collected. Sandfly activity started in end-April and ended in early-November. Mean sandfly density in house was significantly lower than in animal shelters (51 ± 50 *versus* 504 ± 460 sandflies /CDC night, p<0.05). However, a higher proportion of females was found in house (58.4% *versus* 49.2%, p<0.001). Based on species identification of male specimens, *Phlebotomus perniciosus* was the dominant species (56% of the whole male sandfly fauna, p<0.0001). It showed two peaks of density in the active season, a sharp one in early May and a higher long lasting one from end-July to end-September. DNA was extracted from 190 female specimens randomly sampled and corresponding to 96 specimens from house and 94 from animal shelters. Twenty four female sandfly were infected by *Leishmania infantum*. All infected specimens were recognized as *Phlebotomus perniciosus*. *Leishmania infantum* infection rate in female sandflies was 2.3 fold higher in house than in animal shelters (17.7% versus 7.4%, p<0.05). In house, estimated number of infected specimens was the highest at the end of the active season. Abundance, dynamics of density and *Leishmania infantum* infection prevalence of *Phlebotomus perniciosus* in Tunisian hot spot of visceral leishmaniasis highlight the major role of this *Phlebotominae* species in *L*. *infantum* transmission.

## Introduction

Zoonotic visceral leishmaniasis (VL) caused by *Leishmania (L*.*) infantum* is endemic in almost all countries of the Mediterranean basin [1]. The parasite is transmitted by the bite of infected female sandflies belonging to the sub-genus *Larroussius* and *Phlebotomus (P*.*) perniciosus* is one of the major vectors of *L*. *infantum* in the Mediterranean [2–4]. Reservoir hosts are mainly represented by dogs. However, other animals as wild canids, rabbits or hares were also incriminated in *L*. *infantum* transmision [4–7].

In Tunisia, VL remains primarily a pediatric disease that occurs in children less than five years of age [8]. An incidence rate of about 10 VL cases/100,000 children per year is reported for the whole country [9]. However, this incidence rate varies according to geographical area with presence of hot spot region in the Northern part of the country where the disease is homogeneously observed with high frequency [9]. In this interesting location, common ecologic characteristics may have led to uniform high level of disease prevalence.

In his various studies, Rioux conceptualized how ecological and epidemiological concepts and methods could be combined to argument *Leishmania* transmission [10]. Bioclimatic maps were considered as indicators of ecological conditions and were the elective support to identify vector areas and to refer transmission systems to spatial scales [10]. In previous study, we have shown that Tunisian VL hot spot area is located in the semi-arid bioclimatic zone with warm winters and semi-continental climate [9]. We have also reported that *P*. *perniciosus* is the most abundant *Larroussius* species in human leishmaniasis sites located in semi-arid bioclimate [11]. The aim of this work was (i) to provide information about abundance and temporal dynamics of *Larroussius* species in anthropic biotopes of VL hot spot region (ii) to detect *Leishmania* DNA in wild caught female sandflies and (iii) to measure *L*. *infantum* infection rate throughout the active season.

## Materials and methods

### Study area

**Fig 1:**
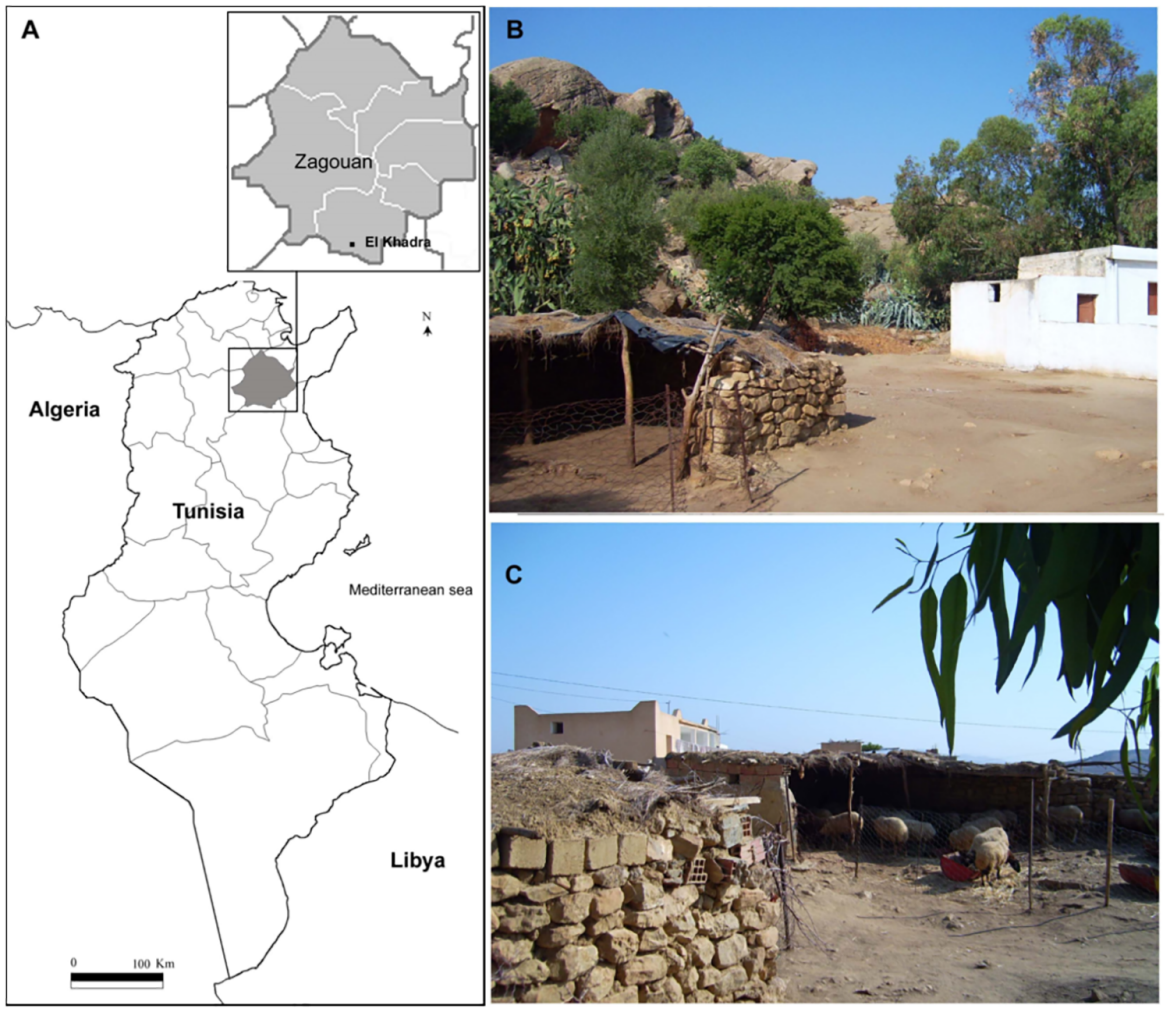
Geographical situation and landscape of sample collection sites. A: The map of Tunisia shows the governorate of Zaghouan (shaded grey) with a focus on the study site location ("El Khadhra"). B and C: two photographs show the house with close semi-open air shelters where are kept animals (mainly sheep). CDC traps were set in house and in animal shelters.

The field study was performed in Eastern-North Tunisia, in Zagouan governorate, on the “dorsale” upland (Fig 1A). The region is locatedin a hot spot area of VL where weather conditions are those typical of the Mediterranean semi-arid climate characterized by typical vegetation series of *Pinus halpensis* and *Tetraclinus articulata*. The sampling station was El khadhra (36°11’05.80”N / 10°02’57.14”E) (Fig 1A). It is a rural locality characterized by the presence of a variety of domestic animals that are potential sandfly hosts with close contact with humans. Animals were either kept in semi-open air shelters built with small tree trunks (mostly goats and sheep) (Fig 1B and 1C) or kept free in at most radius of 100 meters between shelters and houses (mainly dogs, chickens and cats). The study was carried out on private land. Land’s owner gave permission to conduct the study on the site.

### Entomological survey

Sandflies were collected using CDC miniature light traps (John W. Hock Co. FL, U.S.A.) each month, from 26 June 2010 to 12 June 2011 (one year). Two CDC were set up, one inside house (IH) and one in animal shelters (AS) and were operated between 6:00 pm and 08:00 am for one night/month. Over one year, a total of 26 CDC were set up during 13 nights of trapping. No specific permission was required for these activities. The field study did not involve endangered or protected species. Captured specimens were conserved in ethanol 70°. Male (M) specimens were identified at species level according to morphological characters described by Croset *et al*. (1978) [12]. Female (F) specimens were conserved individually for study of *Leishmania* infection.

### Study of *Leishmania* infection

Study of *Leishmania* infection concerned randomly sampled female specimens collected from June to October either in house or animal shelters. Whole bodies of female sandflies were washed twice with distilled water before DNA extraction. DNA was extracted using the method described by Ready *et al*., 1991 [13] with minor modifications. DNA was eluted in 50 μL TE and stored at -20°C until use.

**Fig 2:**
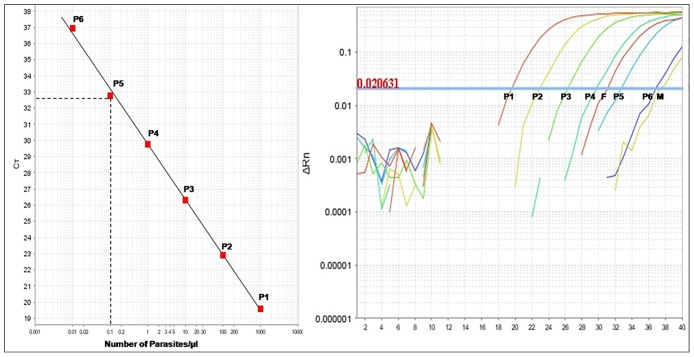
*Leishmania* identification by kDNA real time PCR. A: Standard curve obtained from serial dilutions of *Leishmania* DNA expressed as the number of parasites per reaction tube. The standard curve was established from *Leishmania* DNA extracted from 10^6^ *L*. *infantum* promastigotes. One μl of serial dilutions, ranging from 1000 (P1) to 0.01 parasites (P6) was introduced into reaction tubes. P5 (0.01 parasites per μl) showed a Ct = 32.8. B: Real time PCR amplification curves showing qPCR positive sandfly female specimen (F) as well as negative sandfly male specimen (M). Amplification plots obtained from the serial dilution of *Leishmania* DNA (P1 to P6) are also shown.

All DNA samples were screened for *Leishmania* infection by kinetoplast DNA (kDNA) real-time PCR (qPCR) [14]. Briefly, qPCR was conducted in a final volume of 25 μL by using a TaqMan universal master mixture (Applied Biosystems, USA) containing 100mM of each primer (5’-CTTTTCTGGTCCTCCGGGTAGG-3’) and (5’-CCACCCGGCCCTATTTTACACCAA-3’), 50Mm of probe (FAM 5’-TTTTCGCAGAACGCCCCTACCCGC-3’) and 1 μL of DNA extract. The DNA was amplified in an Applied Biosystems^®^ (Applied Biosystems, Foster City, CA) for 40 cycles at 95 and 60°C. Each sample was tested in duplicate, and each run included both positive and negative controls. The sensitivity of the qPCR reaction was tested by using serial dilutions of parasite DNA extracted from a known number of parasites. Detection of the kinetoplast DNA of *L*. *infantum* reached the level of 0.01 parasites per reaction tube with a dynamic range of 10^5^ (Fig 2A). Real time PCR was considered positive for *Leishmania* when the threshold cycle (Ct) was ≤ to 32.8 which corresponded to 0.1 parasite per reaction tube. Taking into account the amount of biological sample (1 μl of sample DNA) and the elution volume of the extracted DNA (50 μl), selected qPCR threshold corresponded to 5 parasites DNA per sandfly specimen. DNA from 10 male specimens were tested for amplification. Their Ct values were > 36 (Fig 2B). All female specimens that showed a Ct ≤ 32.8 were subsequently assayed by ITS1 PCR.

All positive kDNA qPCR specimens were systematically amplified by ITS1-PCR as previously described [15] using LITSR (5′-CTGGATCATTTTCCGATG-3′) and L5.8S (5′-TGATACCACTTATCGCACTTA-3′) primers. Analysis on a 2% agarose gel was used to verify the amplified product size. ITS1 PCR products were purified by using ExoSAP (ThermoScientific, EU) and *Leishmania* species identification was done by DNA sequencing using an ABI Prism^®^ Big Dye^™^ Terminator, Cycle Sequencing Ready Reaction Kit and AB1 3130 sequencing system (ABI, PE Applied Biosystems), with the same primers used for PCR. DNA sequences from both strands were aligned and edited using Staden software package (http://staden.sourceforge.net/). MEGA version 7 software (www.megasoftware.net) was used to conduct multiple sequence alignments (ClustalW option) and to construct phylogenic tree. Relationships between specimens and reference isolates (*L*. *infantum* isolates from Mediterranean countries as well as Tunisian isolates of *L*. *tropica* and *L*. *major*) were inferred based on genetic distances using the Neighbor Joining (NJ) method using the Kimura 2 parameters model. Statistical support for tree distances was evaluated using bootstrapping (2000 replicates).

### Sandfly molecular typing

Positive female specimens were molecularly typed based on the cytochrome b method. Amplicons were generated by PCR using (5′-CAT/CATTCAA CCA/TGAATGATA-3′) and N1N-PDR (5′-GGTAC/TA/TTTGCCTCGAT/ATTCG T/ATATGA-3′) primers [16]. PCR products were purified and cycle-sequenced as described previously. DNA sequences from both strands were aligned and edited using Staden software package (http://staden.sourceforge.net/). MEGA version 7 software (www.megasoftware.net) was used to conduct multiple sequence alignments (ClustalW option) and to construct phylogenic tree. Sequences were aligned with *Phlebotomus perniciosus* sequences already obtained from Tunisia and other countries [17–19]. Neighbor Joining (NJ) tree was performed using the Kimura 2 parameters model with homologous sequences from *Phlebotomus longicuspis* as the least ambiguous out-group. Statistical support for tree distances was evaluated using bootstrapping (2000 replicates).

### Statistical analysis

Statistical analysis was done using the MedCalc Statistical software (version 11.4.4.0). The t-test was used to compare mean sandfly densities. Chi-squared test was used for comparison of proportions. A test was considered significant if p-value was less than 0.05.

## Results

### Sandfly fauna and seasonal dynamics of male specimens

In one year period, overall 4,441 specimens (2230 males and 2211 females) were collected. Sandfly activity started in end-April and ended in early-November with a mean density of 278 sandflies by CDC night in the active season. Throughout this period mean density in AS (504 ± 460 sandflies / CDC night) was significantly higher than IH (51 ± 50 sandflies / CDC night) (p<0.05). Furthermore, sex-ratio of IH sandflies (0.79) was different from sex-ratio of AS specimens (1.03) with a significant higher proportion of females in house than in animal shelters (58.4% *versus* 49.2%, p = 0.0001) (S1 Table).

Based on species identification of male specimens, *P*. *perniciosus* was the dominant species corresponding to 56% of the whole male sandfly fauna (S1 Table). It was significantly more abundant than other *Phlebotominae* species namely *P*. *papatasi* (32,8%), *Sergentomyia fallax* (3,3%), *Sergentomyia minuta* (2,7%), *P*. *longicuspis* (2,6%), *P*. *antennata* (2.2%) and *P*. *perfiliewi* (0.3%) (p<0.0001).

**Fig 3:**
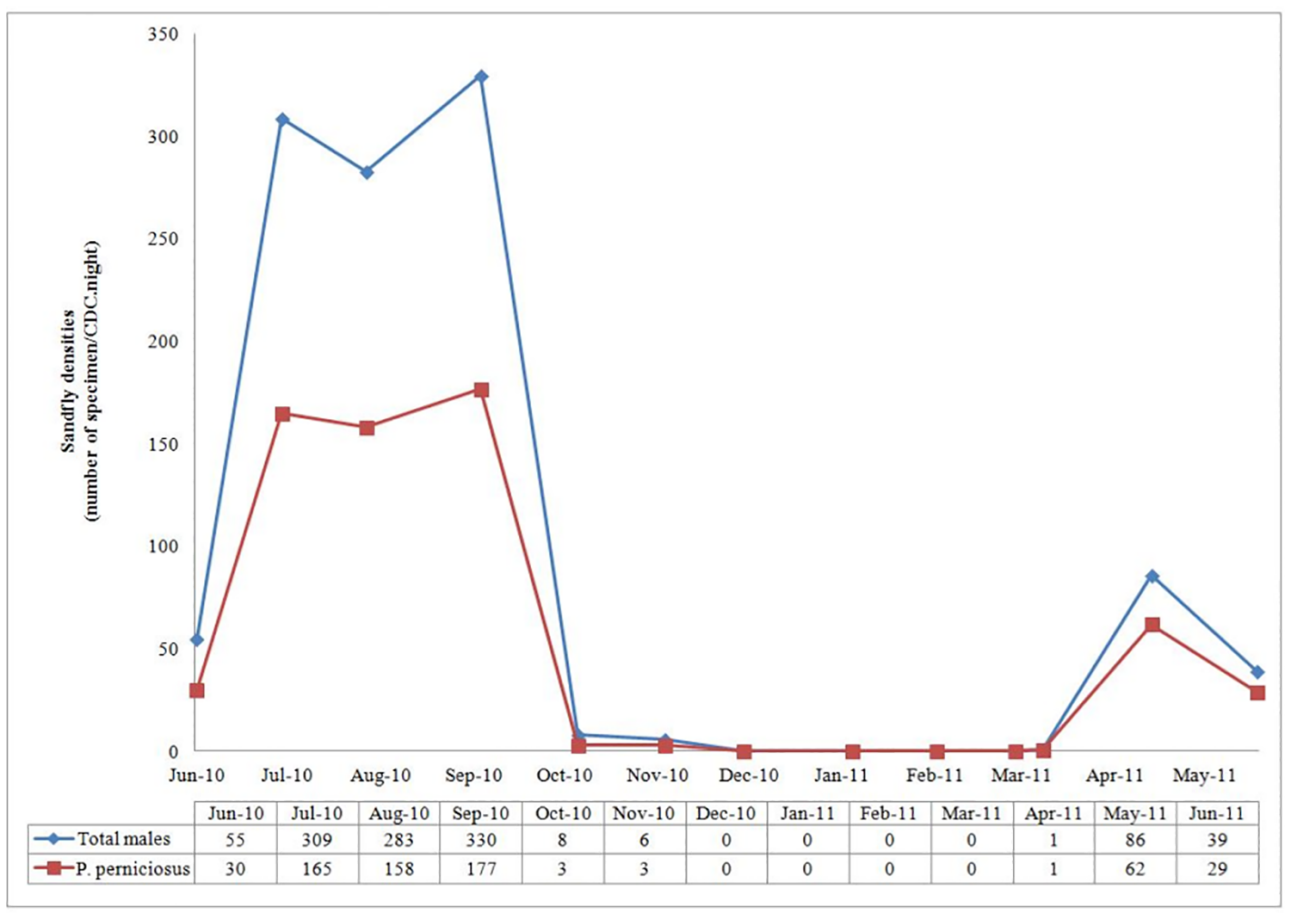
Densities of total sandfly and *Phlebotomus perniciosus* male specimens according to period of capture.

Seasonal density patterns of male specimens were reported in Fig 3. The whole male sandfly fauna as well as the male *P*. *perniciosus* population showed 2 peaks: a sharp one in early May and a higher long lasting one from end-July to end-September (Fig 3).

### *Leishmania* infection in phlebotomine sandfly female specimens

From end-June to end-October, 1715 female specimens (208 from IH and 1507 from AS) were collected and stored individually. According to the period of capture, a dozen to thirty specimens from each monthly IH and AS collection were randomly sampled for DNA extraction (Table 1).

**Table 1:** *Leishmania* infection in phlebotomine female specimens.

	Number of female specimens caught in house			Number female specimens caught in animal shelters		
	Number of collected specimens	Number of DNA extraction	Number of positive ITS1 PCR (infection rate)	Number of collected specimens	Number of DNA extraction	Number of positive ITS1 PCR (infection rate)
**June 26th**	47	23	5 (21.7%)	183	12	1 (8.3%)
**July 24th**	20	8	1 (12.5%)	349	12	1 (8.3%)
**August 21th**	33	33	6 (18.2%)	199	30	5 (16.6%)
**September 28th**	101	25	5 (20%)	713	20	0 (0%)
**October 30th**	7	7	0 (0%)	63	20	0 (0%)
**Total**	208	96	17 (17.7%)	1507	94	7 (7.4%)

**Fig 4:**
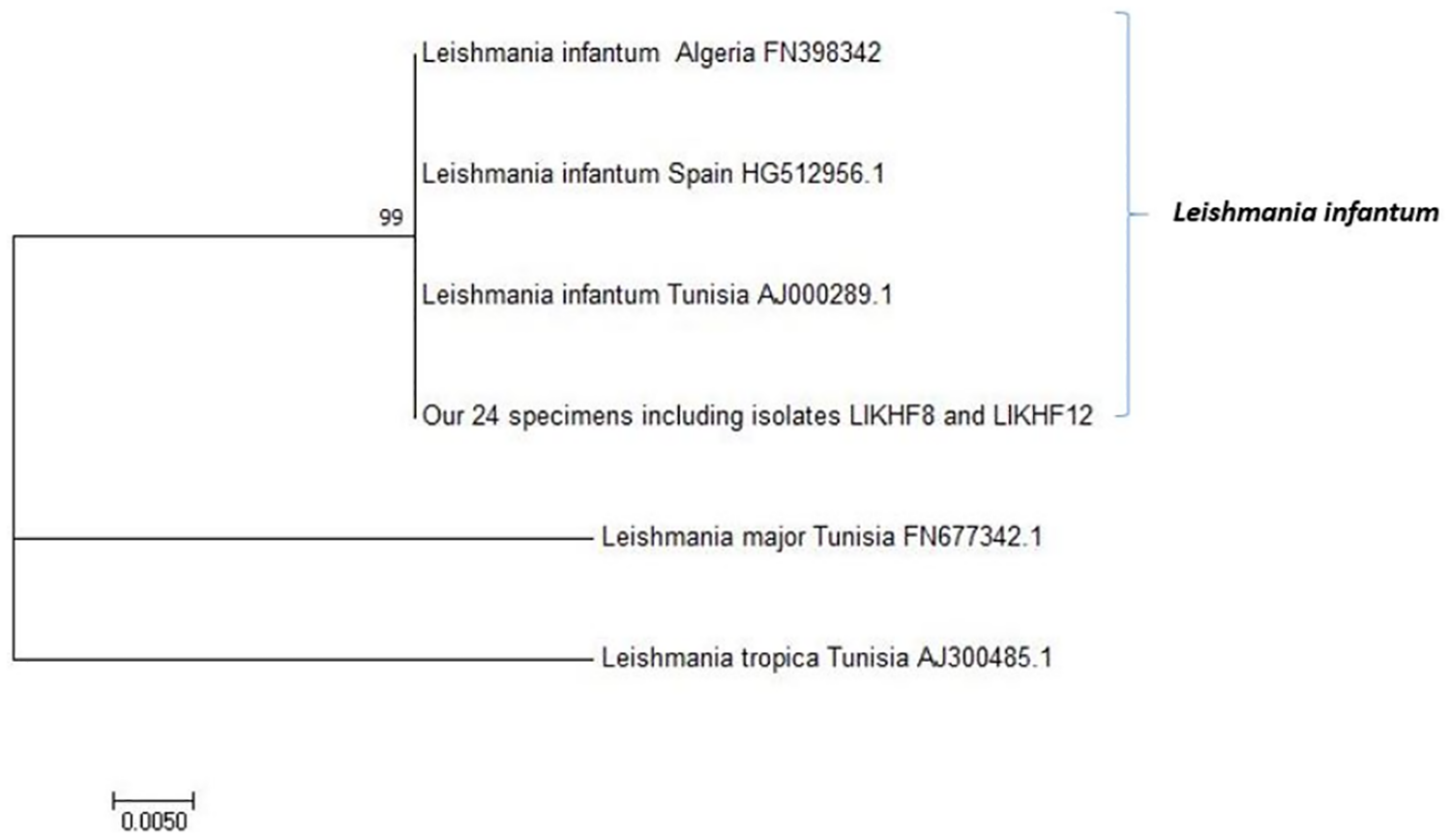
Neighbor-joining tree based on 305 aligned base pairs of the ITS1gene. The sequences from sandflies collected during this study (GenBank accession numbers MF597933 and MF597934) were compared to *L*. *infantum*, *L*. *tropica* and *L*. *major* ITS1 sequences available in GenBank. The percent bootstrap values are indicated on the branches.

Among the 190 analyzed specimens, 28 were positive by kDNA qPCR. Among them, 24 (12.6%) were confirmed by ITS1-PCR and were considered for result analysis (Table 1). ITS1 amplification showed a band of expected size (360 bp). Purified PCR products from the 24 specimens corresponded to one unique sequence identified by inspection of the input data matrix and deposited into GenBank database (http://www.ncbi.nlm.nih.gov/*)* under the accession numbers MF597933 and MF597934. Neighbor Joining analyses positioned our specimens' sequences in the same cluster than *L*. *infantum* sequences reported by other authors (Fig 4). *Leishmania infantum* was retained as the *Leishmania* species infecting female sandflies in the study site.

**Fig 5:**
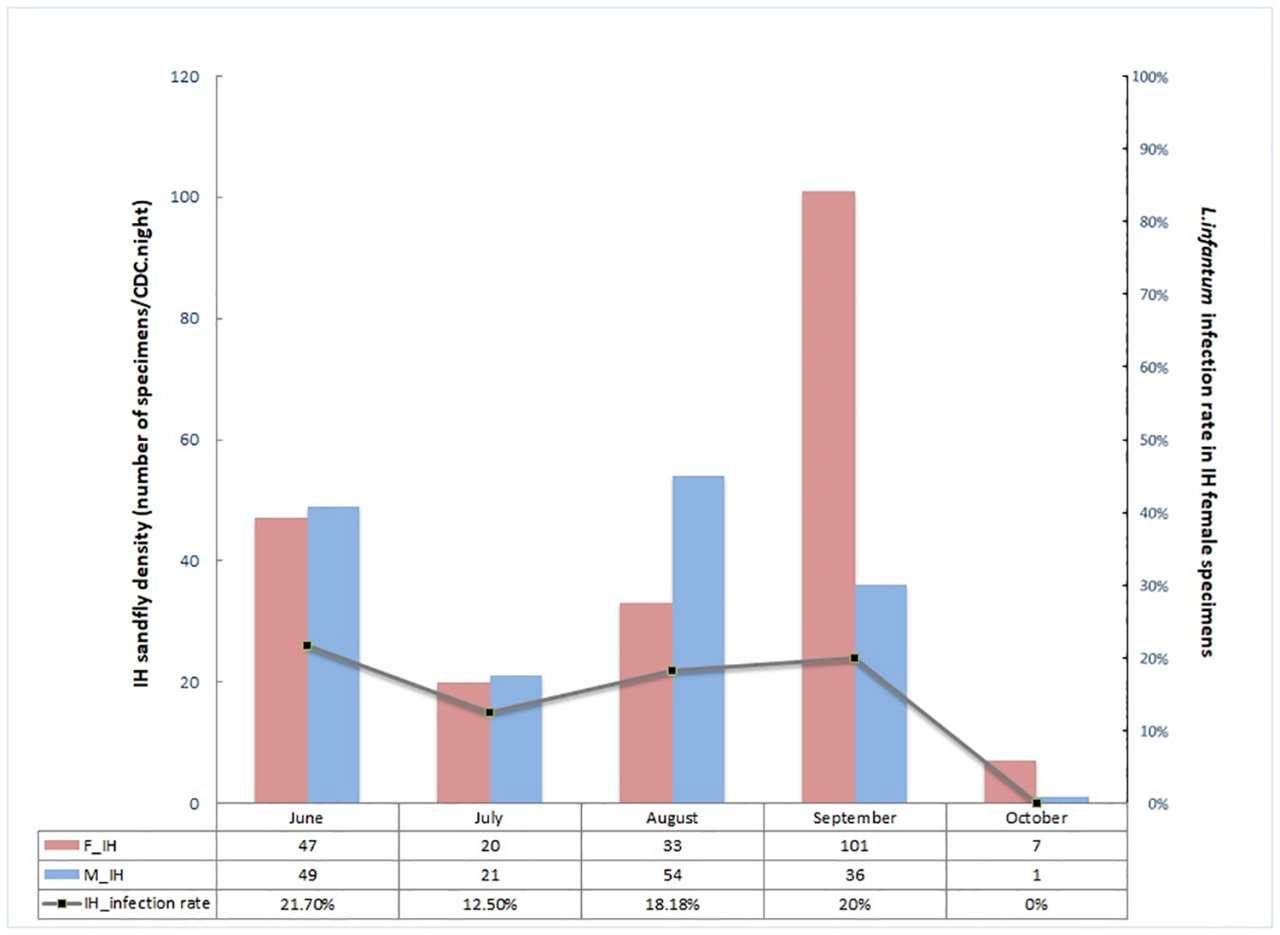
In house male and female sandfly densities and percentage of infected females according to period of capture.

*Leishmania infantum* infection rate in female sandflies was 2.3 fold higher IH than in AS (17.7% versus 7.4%, p<0.05) (Table 1). Fig 5 shows how varied in house the percentage of infected females as well as the density of collected male and female specimens during the period June-October. Male density showed a drastic decrease in July followed by a marked and rapid increase during august. Female density also showed a drastic decrease in July. However, density of female sandfly increased more progressively reaching a pick at the end of September. Percentage of female infection was around 20% in June, dropped to 12.5% in July, increased slowly to about 20% in august and remained steady until late September. Thus, density of female sandfly as well as the estimated number of infected specimens was the highest at the end of the active season. During October, both female density and infection rate decreased significantly (Table 1, Fig 5). Percentage of infected females in animal sheds according to period of sampling is reported in Table 1. The drop of positive sandflies in AS in September should be interpreted with caution given the limited number of specimens explored for infection.

### Sandfly molecular typing

**Fig 6:**
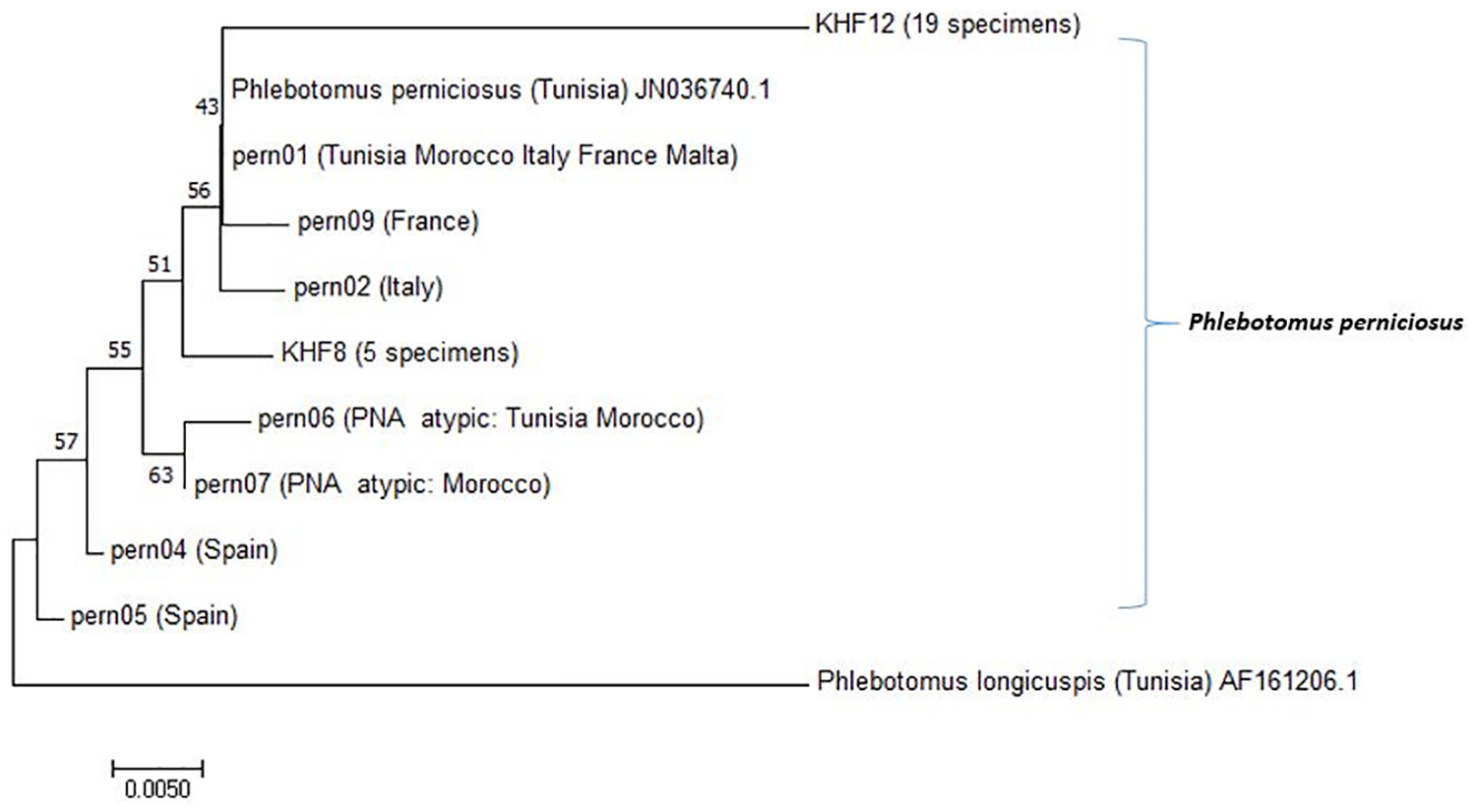
Neighbor-joining tree based on 279 aligned base pairs of the cytochrome b mitochondrial DNA (mtDNA). The sequences from sandflies collected during this study (KHF8 and KHF12) were compared to Mediterranean haplotypes of *P*. *perniciosus* and *P*. *longicuspis* available in GenBank. The percent bootstrap values are indicated on the branches.

For the 24 positive female sandflies infected by *L*. *infantum*, the last 279 nucleotides of Cytochrome b (Cyt b) were sequenced. Two unique sequences (haplotypes KHF8 and KHF12) were identified by inspection of the input data matrix and deposited into GenBank database (http://www.ncbi.nlm.nih.gov/*)*. KHF8 haplotype (accession number MF682976) was identified in 5 specimens. Whereas haplotype KHF12 (accession number MF682977) was shown in 19 specimens. Neighbor joining analyses showed that the two sandfly haplotypes fell in the *P*. *perniciosus* branch. KHF12 which was the predominant haplotype in our study fell in the *P*. *perniciosus* branch that included haplotype pern01 whereas the second haplotye KHF8 defined a new divergent branch (Fig 6). According to place of capture, the 5 infected specimens caught in house in June belonged to the same *P*. *perniciosus* haplotype-KHF8 whereas all positive specimens identified in house since July belonged to the same haplotype-KHF12. In AS, all infected specimens were identified as *P*. *perniciosus* haplotype- KHF12.

## Discussion

Sandflies were monthly collected in anthroponized sites favorable to *L*. *infantum* transmission, where vertebrate hosts including dogs were in close contact to humans. CDC miniature light-traps were used for sandfly collection. They are known to catch active sandflies and then they are useful to determine temporal activity of species [20]. Moreover, they allow to capture phototropic species as *P*. *perniciosus* [21,22].

Seasonal sandfly activity in the study region began in late April and extended to early November. This is in concordance with other entomological studies performed in temperate zones of Mediterranean region, [2,23–25]. In the study site, sandfly density seemed strongly influenced by host abundance and availability [20]. In fact, it was significantly higher in AS then in IH. Moreover, predominance of males in AS may indicate that it can be considered as a sandfly breeding site [26]. Indeed, organic matter produced by domestic animals constitutes suitable conditions for sandfly development [27]. Significant predominance of females in IH may be explained either by their important capacity to disperse [28,29] or by the endophilic behavior of several species present in the site and preferring more closed habitat as houses [30]. Seven species out of the 16 recorded in Tunisia [12,31] were collected. *Phlebotomus perniciosus* was the dominant species as previously described in semi-arid bioclimatic zone [11]. Moreover, this species is known by its ecological plasticity which allows colonization of domestic environment [32].

Phenology of *P*. *perniciosus* showed a typical bimodal distribution as described by previous studies in Tunisia and North Africa [33–35]. The first peak, in early May, is formed by spring generation that emerged from larvae who spent winter in diapauses. Whereas the second long-lasting pick (end-July to end-September) may correspond to more than one wave of emergence in summer [12,34]. On the other hand, *P*. *perniciosus* peaked earlier than described in the same bioclimatic zone [34]. However, surveying population abundance during only one year don’t give reliable picture of abundance patterns of sandfly species and shift of activity can be explained by local climatic events during capturing days [2].

Currently, molecular methods are extensively used to study *Leishmania* infection in wild-caught sandflies [36–39]. kDNA-real time PCR is known to be highly sensitive [40,41] whereas the ITS1 sequencing allows *Leishmania* species identification with high specificity [42]. Using these techniques, we found that *P*. *perniciosus*, the main vector of *L*. *infantum* in Mediterranean basin, was the only infected species by *L*. *infantum* [37,43,44]. Infected *P*. *perniciosus* specimens were already reported in Tunisia [45]. However, *L*. *infantum* transmission in Tunisian foci may involve other *Larroussius* species according to bioclimatic position of the endemic area [46,47].

In the study site, percentage of infected sandfly females was relatively high (12.6%). However, our results correspond to a sandfly collection in very favorable conditions of *L*. *infantum* transmission. In fact, sandflies were caught in a semi-arid bioclimatic zone in peri-domiciliary environment where both *P*. *perniciosus* (the major *L*. *infantum* vector) and dogs (main reservoir hosts) were abundant. In this context, endophilic behavior of *P*. *perniciosus* [30] associated to its high infection rate inside the house support the risk of transmission to human population and may explain the high VL incidence rate in the area.

In house, two picks of sandfly infection were observed, in June and August-September respectively. Each pick of infection concerned a specific *P*. *perniciosus* haplotype and may be linked to a different wave of adult sandfly emergence. In fact, decrease of infection rate observed during July was concomitant to a drop of both male and female sandfly abundance during the same period and was followed by a sharp increase of sandfly density during august suggesting a new wave of emergence (Croset et al, 1970). Furthermore, density of female sandfly as well as the estimated number of infected specimens was the highest at the end of the active season. During this period, females are more likely to be infectious [48].

## Conclusions

In VL hot spot region, *P*. *perniciosus* the main vector of VL in Mediterraneen region was the dominant species. Its population dynamics, endophilic behavior and high infection rate inside houses especially at the end of active season support the risk of transmission to human population. More investigations, especially on reservoir hosts is needed to better document the high VL incidence rate in the area.

## Supporting information

S1 Table Data underlying entomological study. (XLSX)
